# Characterization and immunoprotective efficacy of a fumarate reductase *frdA* mutant of *Salmonella enteritidis*

**DOI:** 10.3389/fmicb.2025.1626276

**Published:** 2025-08-21

**Authors:** Siping Zhu, Xinyi Sun, Hong Li, Yongmei Su, Chihuan Li, Xintong Zhu, Chao Ren, Xiaochen Liu, Yulai Dong, Qiumei Shi, Zhiqiang Zhang

**Affiliations:** ^1^Hebei Key Laboratory of Preventive Veterinary Medicine, Hebei Normal University of Science & Technology, Qinhuangdao, China; ^2^College of Computer Engineering, Zhanjiang University of Science and Technology, Zhanjiang, China; ^3^Weichang Man and Mongolian Autonomous County Xinrui Agricultural Development Ltd., Chengde, China

**Keywords:** *Salmonella enteritidis*, *frdA*, gene deletion, virulence, immune protection

## Abstract

**Background:**

*Salmonella* has the ability to adapt to variable environments by modulating metabolism. The Tricarboxylic Acid Cycle (TCA), as a core metabolic process, is critical for the environmental adaptation and infection process of *Salmonella*. Fumarate reductase FrdA is an important enzyme in the TCA cycle, mainly catalyzing the conversion of fumarate to succinate. But the association between this enzyme and the pathogenicity of *Salmonella* has not yet been reported.

**Methods:**

To determine the role of fumarate reductase FrdA in *Salmonella* infection, a *frdA*-gene deletion strain of *Salmonella enteritidis* (*S. enteritidis*) was generated in this study, and the effect of *frdA* knockout on the biological properties and pathogenicity of *S*. *enteritidis* were further examined. Then, the immunoprotective effect of *frdA*-deficient strain was determined.

**Results:**

The results showed that *frdA* deletion did not affect the growth properties of *S. enteritidis* but caused a significant decreased survival under environmental stress, as well as a substantial decrease in its motility and biofilm formation ability. The Δ*frdA* mutant displayed apparently reduced adhesion and invasion to Caco-2 cells and markedly impaired survival and replication in RAW264.7 cells. The animal infection test showed that the *frdA* gene deletion could lead to a significant decrease in virulence of *S. enteritidis* in mice, with a 64-fold increased LD_50_ for mice, and Δ*frdA* demonstrated significantly decreased colonization in mouse tissues and organs. The transcriptomics results showed that *frdA* deletion resulted in altered expression of 2163 genes in *S. enteritidis*, and downregulated expression of *csgD* and other virulence genes were confimed by qPCR. Moreover, immunization of mice with the *frdA* deletion strain provided promising immune protection for mice.

**Conclusion:**

Fumarate reductase FrdA is closely associated with pathogenicity of *S. enteritidis* and that is an attractive candidate target for vaccine design of *Salmonella*.

## 1 Introduction

It is well-established that bacteria are able to promote infection by shifting their metabolism to adapt to the exposed environment (Encheva et al., 2009; Mitosch et al., 2023). The Krebs cycle, also known as the TCA cycle, is the central part of bacterial metabolism and has been reported to be closely related to the pathogenesis of pathogenic bacteria (Tchawa Yimga et al., 2006). When the bacterial TCA cycle is blocked, it can disturb the overall disruption of bacterial colonization, carbon storage, motility, and host-pathogen interactions (Noster et al., 2019a). As an important zoonotic pathogen, *Salmonella* has to endure diverse and harsh environments, such as digestive tract and barren intracellular environments, in which the regulation and transformation of the metabolic pattern play critical roles (Sun et al., 2023). Previous studies have demonstrated that some drugs may affect the *Salmonella* caused infection by interfering with the TCA cycle, indicating the critical roles of TCA cycle in pathogenicity (Yao et al., 2024; Kim et al., 2025; Chen et al., 2024; Noster et al., 2019a).

The TCA cycle engages multiple enzymes and intermediate metabolites, and it has been documented that the abnormal expression of several key enzymes during bacterial infection suggests that these enzymes are the key factors for TCA to influence bacterial infection (Noster et al., 2019b). The close relationship between these enzymes and metabolites and bacterial virulence was further confirmed by some studies (Ding et al., 2014; Zeng et al., 2020; Himpsl et al., 2020). In some bacteria, some of the TCA-related enzymes have been proven to be excellent vaccine targets (Arora et al., 2018; Altinok et al., 2015).

FrdA is a fumarate reductase encoded by *Salmonella*, an enzyme belonging to the TCA cycle, this enzyme catalyzes the conversion of fumarate to succinate, and it has been reported to greatly increase expression in *Salmonella typhimurium* (Steinsiek et al., 2011; Noster et al., 2019b; Westerman et al., 2021), but its exact role in *Salmonella*-caused infection is still unclear. In the present study, we constructed a *frdA* gene deletion strain of *S. enteritidis* and attempted to study the effect of FrdA on the virulence of *S. enteritidis* and assessed its immunoprotective potential as a vaccine.

## 2 Materials and methods

### 2.1 Bacterial strains, cells, and plasmids

*S. enteritidis* C50336 was isolated from the feces of a patient with diarrhea and purchased from the National Institute for the Control of Pharmaceutical and Biological Products (China). It was kept in the Key Laboratory of Preventive Veterinary Medicine, Hebei Province. All strains were cultured in Luria broth (LB) medium Haibo Biotechnology Co., Ltd., HB0128, China) at 37°C, and added ampicillin (100 μg/mL) or chloramphenicol (34 μg/mL) as required. The Caco-2 BBE cells and RAW264.7 cells were purchased from BeNa Culture Collection (Shanghai, China) and cultured in Dulbecco's Modified Eagle Medium (DMEM) (Thermo Fisher Scientific Co., Ltd., TFS12491023, China) containing 10% fetal bovine serum (Thermo Fisher Scientific Co., Ltd., China) at 37°C in an incubator with 5% CO_2_. The plasmids pKD3, pKD46, pBR322 and pCP20 for bacterial gene knockout were provided by Invitrogen.

### 2.2 Experimental animals

Female Kunming mice aged 6–8 weeks were purchased from Beijing Speifu Biotechnology Co., Ltd. (Beijing, China).

### 2.3 Construction of *S. enteritidis frdA*-deficient mutant and complemented strains

The λ-Red homologous recombination technique was used to knock out the *frdA* gene (Zhang et al., 2020). Briefly, the chloramphenicol resistance cassette (cat) was amplified using long primers P1 and P2 (Table 1), which contain homologous fragments of the *frdA* gene, with plasmid pKD3 as a template. The purified PCR product was electrotransferred into the competent cell of the C50336 strain (harboring plasmid pKD46) to obtain a primary recombinant strain Δ*frdA*::*cat*. The inserted cat gene was eliminated by further electrotransferring the pCP20 plasmid. The obtained strain was then placed in a water bath and treated at 42°C for 5–6 h to remove the temperature-sensitive plasmid pCP20. The *frdA* deletion was confirmed by PCR with primers P3 and P4 (Table 1). Subsequently, the purified PCR product was cloned into pMD-19T vector (Takara Biomedical Technology (Beijing) Co., Ltd.), and the recombinant vector was sent to Sangon Biotech (Shanghai) Co., Ltd. (China) for sequencing.

**Table 1:** PCR primer information.

**Primers**	**Nucleotide sequences (5^′^-3^′^)**	**Amplification product size (bp)**
P1 (KO-*frdA*F)	CTGCCGCACAGGCGAATCCCAATGCTA	760
	AAATCGCACTGATCTCAAAAGTGTACCC	
	GATGTGTGTAGGCTGGAGCTGCTTCG	
P2 (KO-*frdA*R)	GCCACGTTCAGACCATGACCCAGTTCGAT	
	GGTATACAGCAGGTCAGTGTTGAACACGC	
	TCATATGAATATCCTCCTTAG	
P3 (ID-*frdA*F)	GCATTCATACTTCGCAGAACCCA	1,950/760^**^
P4 (ID-*frdA*R)	CCGCTTCACCGCCGTAAACAC	
P5 (RS-*frdA*F)	CGGGATCCGCCTTCTGGAGGGTAAAAAAAGTGA	760
P6 (RS-*frdA*R)	ACGCGTCGACTCAGCCATTCGCCTTCTCCTTCT	
P7 (*frdAF)*	CATACCCTGTTCCAGACTTCCC	122
P8 (*frdAR)*	TCCATCATGTTCATTGCCACC	

^**^Stand for C50336Δ*frdA*.

To generate the complemented strain, the whole ORF of *frdA* was amplified using primers P5 and P6 (Table 1) with the genome of the C50336 strain as a template. The PCR product was cloned into the pBR322 plasmid by using *Bam*HI (NEB #R3136) and *Sal*I (NEB #R3138) nucleic acid endonuclease (Takara Biomedical Technology (Beijing) Co., Ltd.,). The constructed pBR322-*frdA* plasmid was then transfered into the Δ*frdA* mutant, with positive clones verified by PCR using primers P3 and P4, and named Δ*frdA*+*frdA*. The *frdA* gene expression in Δ*frdA* and Δ*frdA*+*frdA* was confirmed by qPCR. Briefly, RNA of each strain was extracted using a bacterial RNA extraction kit (Beijing Aidlab Biotechnologies Co., Ltd., RN63, China), reverse transcribed into cDNA, and subjected to qPCR verification using primers P7 and P8 (Table 1) to assess the expression of the *frdA* gene.

### 2.4 Stress tolerance assay

Bacteria were cultured to the logarithmic growth phase and diluted to 10^7^ CFU/mL with saline. The bacterial suspension was separately incubated inacidic saline (pH 4.0) and alkaline saline (pH 10.0) at a ratio of 1:100, treated for 1 h, and then doubly diluted with phosphate-buffered saline (PBS) (Thermo Fisher Scientific Co., Ltd., TFS20012050, China) for bacteria counting. For heat stress, the bacterial suspension was incubated in PBS at a ratio of 1:100 and then treated under 42°C for 1 h, and for oxidative stress, the bacterial suspension was incubated in PBS containing 10 mmol/L H_2_O_2_ and treated for 10 min. The bacterial suspension after treatment under heat and oxidative stress were doubly diluted with PBS for bacteria counting. Bacteria were counted before and after the stress treatment, and the survival rate under stress conditions was calculated by dividing the bacteria count post-treatment by the bacteria count pre-treatment (Zhang et al., 2024).

### 2.5 Biofilm formation assay

The biofilm formation ability of each strain was determined using crystalline violet (CV) staining (Simm et al., 2014). Briefly, each bacterial strain was inoculated in LB and incubated statically at 30°C for 3 days. The bacterial culture was removed, and the formed biofilm was washed 3 times with PBS and fixed in methanol (Tianjin Fuyu Fine Chemical Industry Co., Ltd., 67-56-1, China) for 10 min, followed by staining using 2% CV (Shanghai Macklin Biochemical Co., Ltd., MFCD00011750, China) for 15 min for observing the thickness and color of the biofilm ring on the glass tubes. To quantify the biofilm formation, each strain was inoculated in a 96-well plate (Guangzhou Jet Bio-Filtration Co., Ltd., TCP011896, China) in 100 μL of LB and incubated statically at 30°C for 3 days. CV staining of the biofilm was carried out as above. The biofilm-bound CV was dissolved in 100 μL of ethanol (Tianjin Fuyu Fine Chemical Industry Co., Ltd., 64-17-5, China) and subjected to determination of the absorbance under 570 nm (OD_570_). The assay was repeated 3 times.

To investigate the expression of the main components of biofilm, curli fimbriae and cellulose, we inoculated each strain on Congo red (Tianjin Damao Chemical Reagent Partnership Enterprise (Limited Partnership), AR3749, China) and Coomassie brilliant blue plate (Beijing Solarbio Science & Technology Co., Ltd., C8420, China) or LB agar containing Calcofluor White Stain (200 mg/L) without salt, respectively. As previously reported (Zhao et al., 2024), five μL of bacterial culture of each strain and inoculated it onto LB agar containing 160 mg/L Congo red and 10 mg/L Coomassie brilliant blue without salt and incubated at 30°C for 2 days, and the colony morphology and color were observed to assess the production of curli fimbriae. Then, five μL of bacterial culture of each strain was inoculated onto LB agar containing Calcofluor White Stain (200 mg/L) without salt and incubated at 30°C for 2 days, and the colony morphology was observed under ultraviolet (UV) light (366 nm) to determine the production of cellulose.

### 2.6 Motility assay

The motility assay of each strain was assayed by determining the bacterial range formed on semi-solid media. Five μL of bacterial culture of each strain was inoculated onto semi-solid LB plates containing 0.3% agar and incubated at 37°C for 6–8 h. The diameter of the bacterial zone was measured, and the assay was repeated 3 times.

### 2.7 Adhesion, invasion, and intracellular survival assays

The adhesion and invasion abilities of each strain were assayed via a Caco-2 cell model. The Caco-2 cells were seeded in a 6-well plate (Guangzhou Jet Bio-Filtration Co., Ltd., TCP001006, China), and the bacterial suspensions of logarithmic phase were added into the cell wells with a multiplicity of infection (MOI) of 100 (Zhou et al., 2025). The cell plates were centrifuged at 1,000 rpm for 5 min and then incubated for 1 h. The cells were washed with PBS three times to remove the free-standing bacteria. Then the cells were lysed with 1% Triton X-100 (Beijing Solarbio Science & Technology Co., Ltd., T8200, China) and the lysates were serially diluted for bacterial counting. The bacterial adhesion rate was calculated by dividing the number of adherent bacteria by that of the initially added bacteria (Zhang et al., 2020). For the invasion assay, preliminary operations were the same as the adhesion test, and at 1 h after infection, the cells were continually incubated in DMEM containing gentamicin (100 μg/mL) for 1 h to kill the extracellular bacteria. Then, the cells were lysed for bacteria counting. The invasion rate was calculated by dividing the number of invasive bacteria by that of the initially added bacteria (Zhang et al., 2020).

Intracellular survival assays were assayed in RAW264.7 cells. The former operation was the same as that of the invasion experiment. The RAW264.7 cells were seeded in a 6-well plate and infected with bacteria of logarithmic phase at an MOI of 100. The cells were cultured in DMEM containing gentamicin (100 μg/mL) after adhesion for 1 h and then lysed for bacteria counting at 3 and 23 h post-infection (hpi). Intracellular survival rate = (Number of bacteria inside cells at 23 hpi/Number of bacteria inside cells at 3 hpi) × 100% (Zhang et al., 2020).

### 2.8 RNA extraction, sequencing, and bioinformatics analyses

Three biological replicates of C50336 strain and the *frdA* mutant were cultured to an OD_600_ of 0.6 in LB medium. Total RNA of each strain was extracted using an RNA extraction kit (Beijing Aidlab Biotechnologies Co., Ltd., China) and the residual genomic DNA is removed by DNase treatment. The concentration, purity and integrity of RNA samples were quantified by a UV spectrophotometer and a Bioanalyzer instrument. RNA-seq assay was performed using the Majorbio Cloud platform (www.majorbio.com), and each sequencing library was generated using the TruSeqTM RNA sample preparation Kit (Illumina, Inc., CA). Differentially expressed genes (DEGs) were identified as the genes with a fold-change (treatment/control) of >2 or <0.5 and a corrected *p*-value <0.05.

### 2.9 RNA extraction and quantitative real-time PCR (qPCR)

qPCR was preformed to assess the expression of bacterial virulence gene. RNA was extracted using a bacterial RNA extraction kit (Beijing Aidlab Biotechnologies Co., Ltd., China) and treated with DNase I to remove genomic DNA. Then, RNA was taken as template to produce cDNA using a reverse transcription kit (Bohang Biotechnology Co., Ltd., China). The SYBR Green dye based qPCR was performed using this cDNA as template as described previously (Nguyen et al., 2021). The primers used were illustrated in Table 2.

**Table 2:** qPCR primer information.

**Primers**	**Nucleotide sequences (5^′^-3^′^)**
flgGF	GCGCCGGACGATTGC
flgGR	CCGGGCTGGAAAGCATT
invHF	CCCTTCCTCCGTGAGCAAA
invHR	TGGCCAGTTGCTCTTTCTGA
hflKF	AGCGCGGCGTTGTGA
hflKR	TCAGACCTGGCTCTACCAGATG
ssrAF	CGAGTATGGCTGGATCAAAACA
ssrAR	TGTACGTATTTTTTGCGGGATGT
orf245F	CAGGGTAATATCGATGTGGACTACA
orf245R	GCGGTATGTGGAAAACGAGTTT
prot6EF	GAACGTTTGGCTGCCTATGG
prot6ER	CGCAGTGACTGGCATCAAGA
rfbHF	ACGGTCGGTATTTGTCAACTCA
rfbHR	TCGCCAACCGTATTTTGCTAA
sipBF	GCCACTGCTGAATCTGATCCA
sipBR	CGAGGCGCTTGCTGATTT
ompRF	TGTGCCGGATCTTCTTCCA
ompRR	CTCCATCGACGTCCAGATCTC
sodCF	CACATGGATCATGAGCGCTTT
sodCR	CTGCGCCGCGTCTGA
sipAF	CAGGGAACGGTGTGGAGGTA
sipAR	AGACGTTTTTGGGTGTGATACGT
ssaVF	GCGCGATACGGACATATTCTG
ssaVR	TGGGCGCCACGTGAA
pipBF	GCTCCTGTTAATGATTTCGCTAAAG
pipBR	GCTCAGACTTAACTGACACCAAACTAA
spvBF	TGGGTGGGCAACAGCAA
spvBR	GCAGGATGCCGTTACTGTCA
Primers	Nucleotide sequences (5′-3′)
xthAF	CGCCCGTCCCCATCA
xthAR	CACATCGGGCTGGTGTTTT
mgtCF	CGAACCTCGCTTTCATCTTCTT
mgtCR	CCGCCGAGGGAGAAAAAC
mrr1F	CCATCGCTTCCAGCAACTG
mrr1R	TCTCTACCATGAACCCGTACAAATT
csgDF	GCCTCATATTAACGGCGTG
csgDR	AGCGGTAATTTCCTGAGTGC
bcsAF	GCCCAGCTTCAGAATATCCA
bscAR	TGGAAGGGCAGAAAGTGAAT
csgAF	AATGCCACCATCGACCAGTG
csgAR	CAAAACCAACCTGACGCACC
tatAF	AGTATTTGGCAGTTGTTGATTGTTG
tatAR	ACCGATGGAACCGAGTTTTTT
16SF	CCAGGGCTACACACGTGCTA
16SR	TCTCGCGAGGTCGCTTCT

### 2.10 Determination of LD_50_ in mice

Kunming (KM) mice were used for determine the LD_50_ of each strain. One hundred and ten female KM mice were randomly divided into 11 groups with 10 mice per group. The five groups were intraperitoneally (i.p.) injected with C50336 at doses ranging from 2 × 10^9^ to 2 × 10^5^ CFU/mouse. Another five groups were i.p. injected with Δ*frdA* at doses ranging from 1 × 10^9^ to 1 × 10^5^ CFU/mouse. And the left one group was intraperitoneally injected with an equal volume of PBS as a control. The mice were observed and recorded for abnormal performance and death for 14 days. The LD_50_ value was calculated using the Modified Karber method (Park et al., 2020).

### 2.11 Bacterial load assay in tissues and organs of mice

Forty-five female KM mice were divided into three groups, with 15 mice in each group. Each group was intraperitoneally injected with a bacterial suspension of C50336, Δ*frdA*, or PBS at a dose of 1 × 10^5^ CFU/mouse. The mice were euthanized under anesthesia at different time points (3–14 days), and the spleen, liver, and lungs were aseptically picked and homogenized for bacteria counting on *Salmonella*-Shigella (SS) agar (Beijing Auboxing Biotechnology Co., Ltd., AUB02-003, China).

### 2.12 Determination of immunoprotective potential

Thirty KM mice (6–8 weeks old) were randomly divided into three groups, named immunized, unimmunized, and control groups, respectively. For the immunized group, mice were i.p. injected with a dose of 1 × 10^6^ CFU/mouse of Δ*frdA* (once on day 0 and once on day 14), and the other two groups were intraperitoneally injected with an equal amount of sterile PBS. At 28 days post-immunization, the mice of immunized and unimmunized were intraperitoneally injected with a lethal dose of strain C50336, at 2 × 10^7^ CFU/mice, whereas the control group received PBS. The mortalities were recorded every day for 14 days, and the relative percentage of survival (RPS) was calculated as [1 – (mortality in Δ*frdA* immunization group/mortality in challenge group)] × 100% (Park et al., 2020).

### 2.13 Determination of serum antibody IgG level in mice

Forty KM mice (6–8 weeks old) were randomly divided into two groups, named immunized and control groups, respectively. Immunization was carried out according to the method of 2.11; at the 0th, 7th, 14th, 21st, and 28th d of immunization, blood was collected from the tail tip of three mice in each group at random, and the blood was centrifuged at 3,000 rpm for 5 min. The serum antibody IgG levels of the two groups of mice were determined by indirect ELISA method (Zhao et al., 2025).

### 2.14 Determination of spleen index in mice

Thirty KM mouse (6–8 weeks old) were randomly divided into two groups, named immunized and control groups, respectively. Immunization was carried out according to the method of 2.11. At 3 d, 7 d, 14 d, and 21 d of immunization, three mice were randomly selected in each group, and the spleens of the mice were removed and weighed to calculate the spleen index. Spleen index = [spleen weight (g)/mouse body weight (g)] × 100 % (Arunima et al., 2020).

### 2.15 Determination of transformed proliferative capacity of mouse lymphocytes

Mice (*n* = 3) from the control and immunized groups at 14 days post immunization were euthanized. The spleens were aseptically collected, and the lymphocytes were separated by homogenization and filtration via a 70 μm cell strainer. After cell counting, lymphocytes were seeded into 96-well tissue culture plate at 5 × 10^5^ cells/well. The cell wells were added with ConA (Concanavalin A) (final concentration of 5 μg/mL) (Shanghai Biyuntian Biological Co., Ltd., ST2062, China), C50336 bacterial antigen (final concentration of 5 μg/mL), and RPM1640 as control, respectively. The plate was incubated for 72 h at 37°C. 3-(4,5-dimethylthiazol-2-yl)-2,5-diphenyltetrazolium bromide (MTT) solution (5 mg/mL, Shanghai Biyuntian Biological Co., Ltd., ST316, China) was added, incubated for 4 h (Dong et al., 2011), followed by the addition of Formazan solvent (Shanghai Biyuntian Biological Co., Ltd., ST316, China), and incubated for another 4 h for measuring OD_570_. The stimulation index (SI) was calculated according to the formula: [SI = (OD value of stimulated group – OD value of culture medium group)/(OD value of unstimulated group – OD value of culture medium group)] (Dong et al., 2011).

### 2.16 Ethics statement

All animal experiments were conducted in full compliance with international ethical standards and the Experimental Animal Regulation Ordinances (HPDST 2020-17) as stipulated by the Hebei Provincial Department of Science and Technology. The study protocol was reviewed and approved by the Animal Care and Use Committee of Hebei Normal University of Science and Technology.

### 2.17 Statistical analysis

Statistical analyses were performed using GraphPad Prism version 9.5.0, with the one-way Analysis of Variance (ANOVA) followed by *t*-tests. Data were expressed as mean ± standard error. Significant differences were denoted with an asterisk (^*^), where ^*^p < 0.05, ^**^p < 0.01, and ^***^p < 0.001 are considered to represent statistically significant differences in mean values.

## 3 Results

### 3.1 The *frdA* gene deletion does not affect the growth of *S. enteritidis* in LB

**Figure 1:**
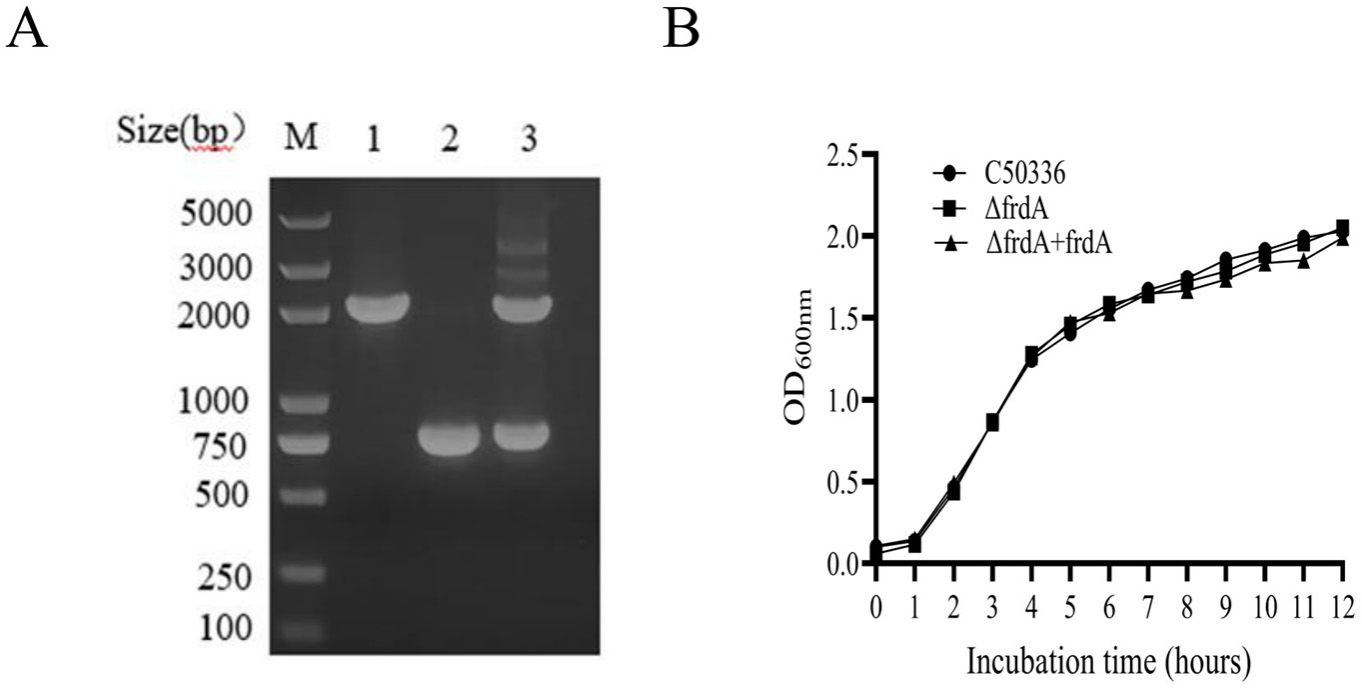
**(A)** PCR verification of the *frdA* gene deletion strain. Lane M: DL2000 DNA Marker (Takara). Numbers 1 means the wild-type strain (C50336); Numbers 2 means Δ*frdA*-deletion strain; Numbers 3 means Δ*frdA*-complemented strain. The PCR product of C50336 has a length of 1,950 bp, the product of Δ*frdA*-deletion strain has a length of 760 bp, the product of Δ*frdA*-complemented strain has a length of 1,950 bp. **(B)** The results of growth curve of 3 strains of bacteria were determined. The absorbance of C50336, Δ*frdA* and Δ*frdA* + *frdA* bacterial fluids was measured at 600 nm every 1 h, and the growth curves were plotted for 12 h consecutively.

Using λ-Red recombination technology, a *frdA* gene deletion mutant of *S. enteritidis* C50336 and complemented strain were constructed. The *frdA* knockout mutant and the complemented were confirmed by PCR (Figure 1A).

To access the influence of *frdA* deletion on the growth of *S. enteritidis*, we examined the growth of each strain in LB medium. The data showed (Figure 1B) that all three stains displayed similar growth curves in LB, demonstrating that *frdA* deletion does not affect the growth of *S. enteritidis* in LB.

### 3.2 The *frdA* gene affects the tolerance of *S. enteritidis* to stress conditions

**Figure 2:**
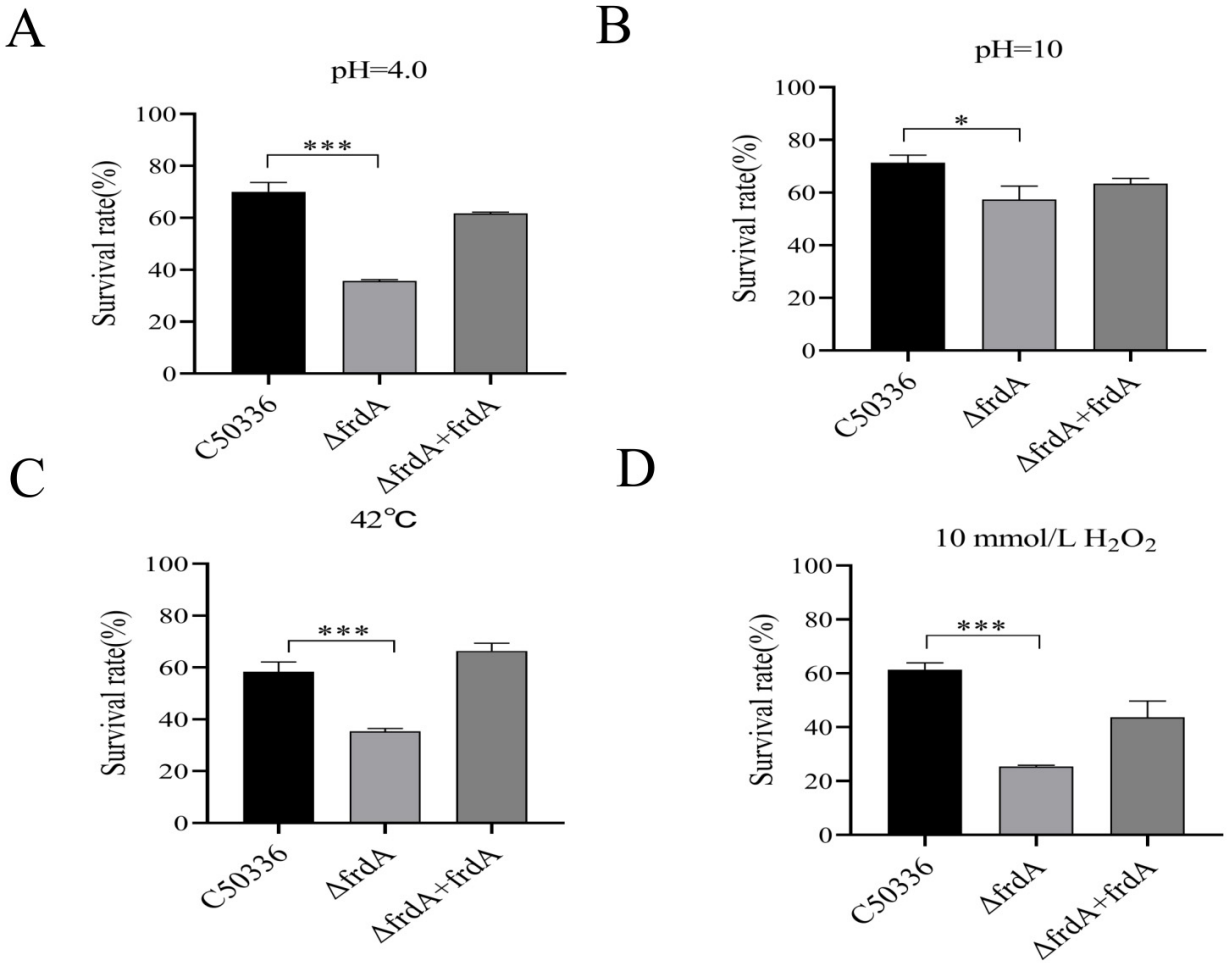
The survival rate of Δ*frdA* under various environmental stresses. **(A)** Acidic stress. **(B)** Alkaline stress. **(C)** Heat stress. **(D)** Oxidative stress. The data represents the average of 3 replicates (*p < 0.05, ***p < 0.001).

To investigate whether the *frdA* gene affects the resistance of *S. enteritidis* to various environmental stresses, we compared the survival of C50336, Δ*frdA*, and Δ*frdA* + *frdA* under conditions of acid solution, alkaline solution, heat stress and oxidative stress. The results showed a significantly decreased survival of the Δ*frdA* strain under acidic (Figure 2A), alkaline (Figure 2B), heat stress (Figure 2C), and oxidative stress (Figure 2D) conditions as compared to the C50336 and Δ*frdA* + *frdA* strains. These results suggest that the *frdA* gene plays important roles in the resistance of *S. enteritidis* to acid, alkali, heat stress, oxidative stress, and nitrification stress.

### 3.3 The *frdA* gene knockout leads to reduced biofilm formation of of *S. enteritidis*

**Figure 3:**
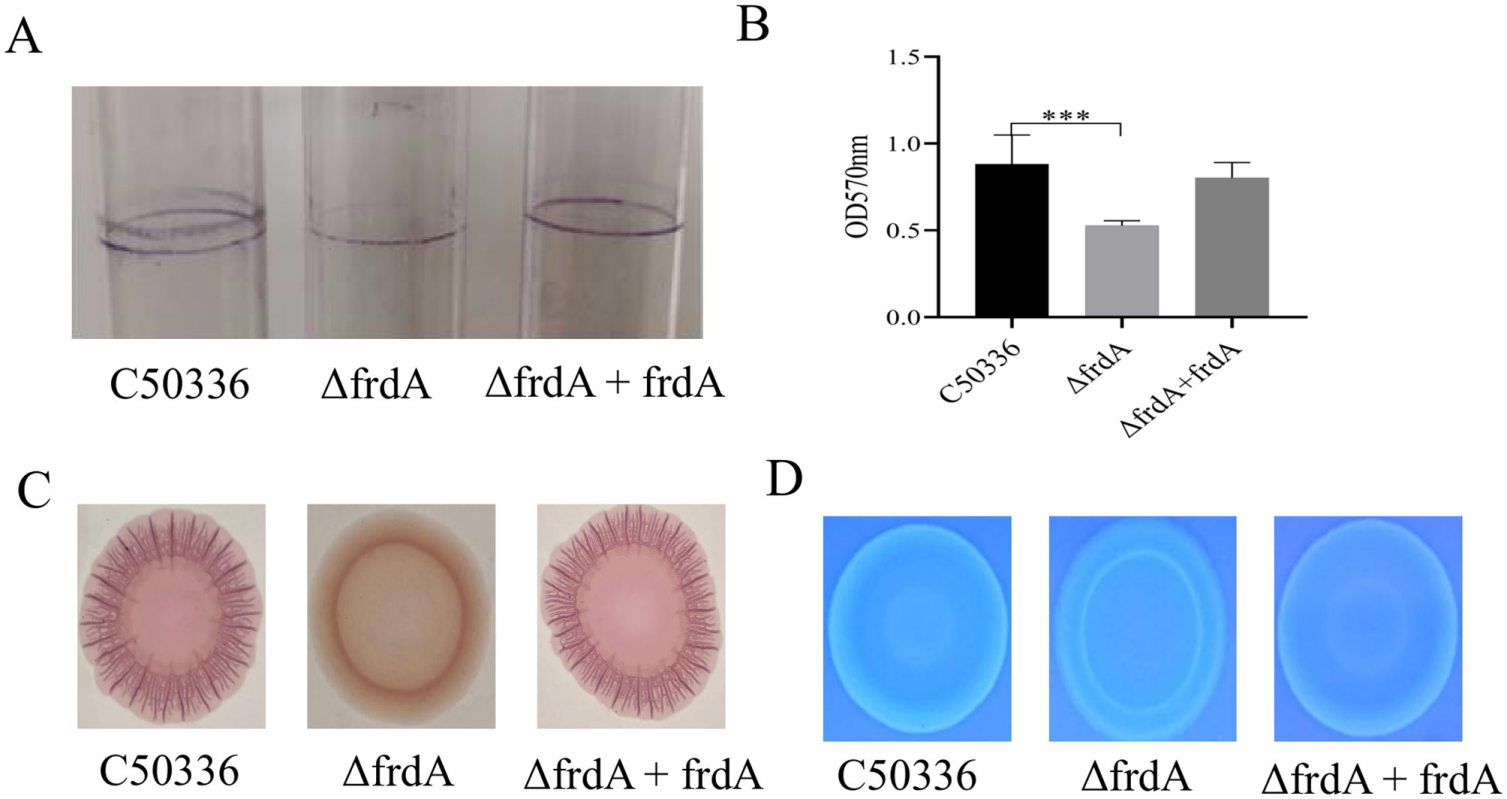
**(A)** Detection of biofilm formation in glass test tubes. **(B)** Quantitative detection of biofilm formation in microtiter plates, with absorbance measured at 570 nm. **(C)** Curli formation detection. **(D)** Cellulose formation detection. ***p < 0.001.

The biofilm formation ability of C50336, Δ*frdA*, and Δ*frdA* + *frdA* was examined in the present study, and the data showed that the Δ*frdA* displayed apparently impaired biofilm formation compared with C50336 and Δ*frdA* +*frdA* (Figure 3A). Quantitative results revealed that the biofilms formed by the Δ*frdA* strains was significantly different at OD_570nm_ after staining and dissolution (Figure 3B). The Δ*frdA* formed a colony with fewer wrinkles and a lighter color on the Congo red medium as compared with C50336 and Δ*frdA* + *frdA* (Figure 3C), indicating that the deletion of *frdA* reduced curli production. The colony of Δ*frdA* showed much weaker fluorescence under UV light (Figure 3D), suggesting the decreased production of cellulose. Taken together, all these results demonstrated that *frdA* is associated with the biofilm formation of *S. enteritidis*.

### 3.4 The *frdA* gene is associated with motility of *S. enteritidis*

**Figure 4:**
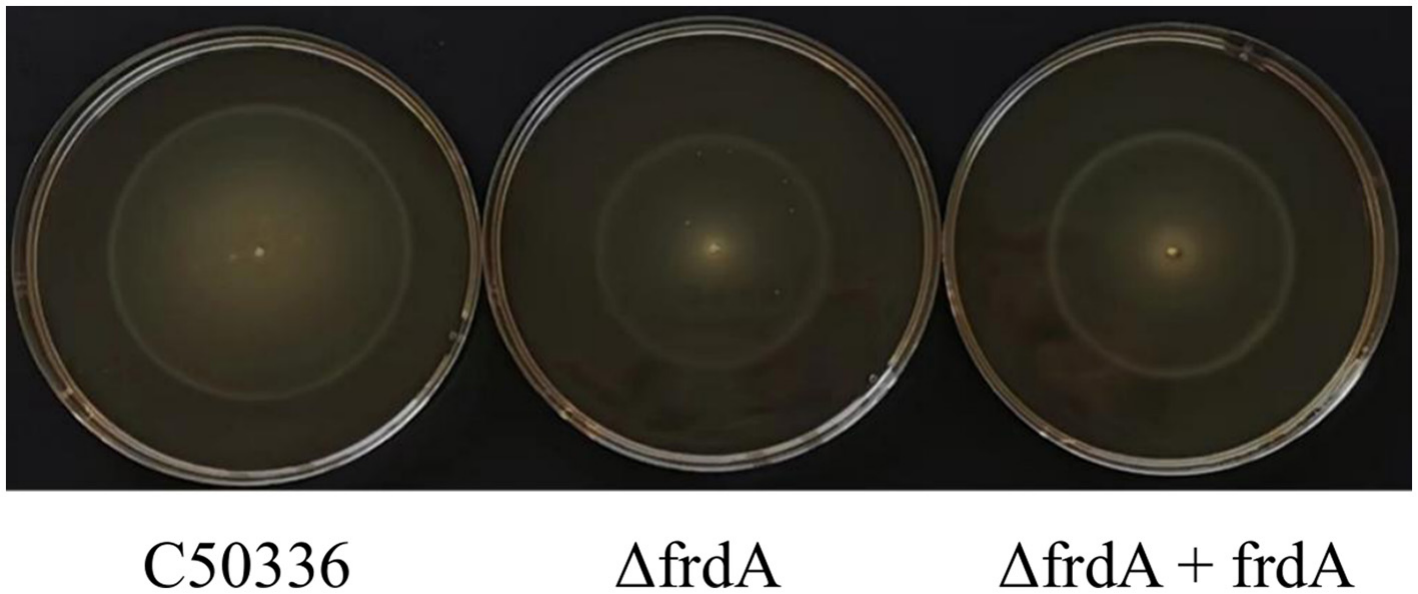
The motility of the strains was evaluated on 0.3% agar plates, measured after 5 h of incubation.

The motility of C50336, Δ*frdA*, and Δ*frdA* + *frdA* was assessed on semi-solid medium. The Δ*frdA* showed much smaller bacterial zone on semi-solid plate as compared with C50336 and Δ*frdA* +*frdA* in present study (Figure 4).

### 3.5 The *frdA* gene affects the adhesion, invasion and intracellular survival ability of *S. enteritidis*

**Figure 5:**
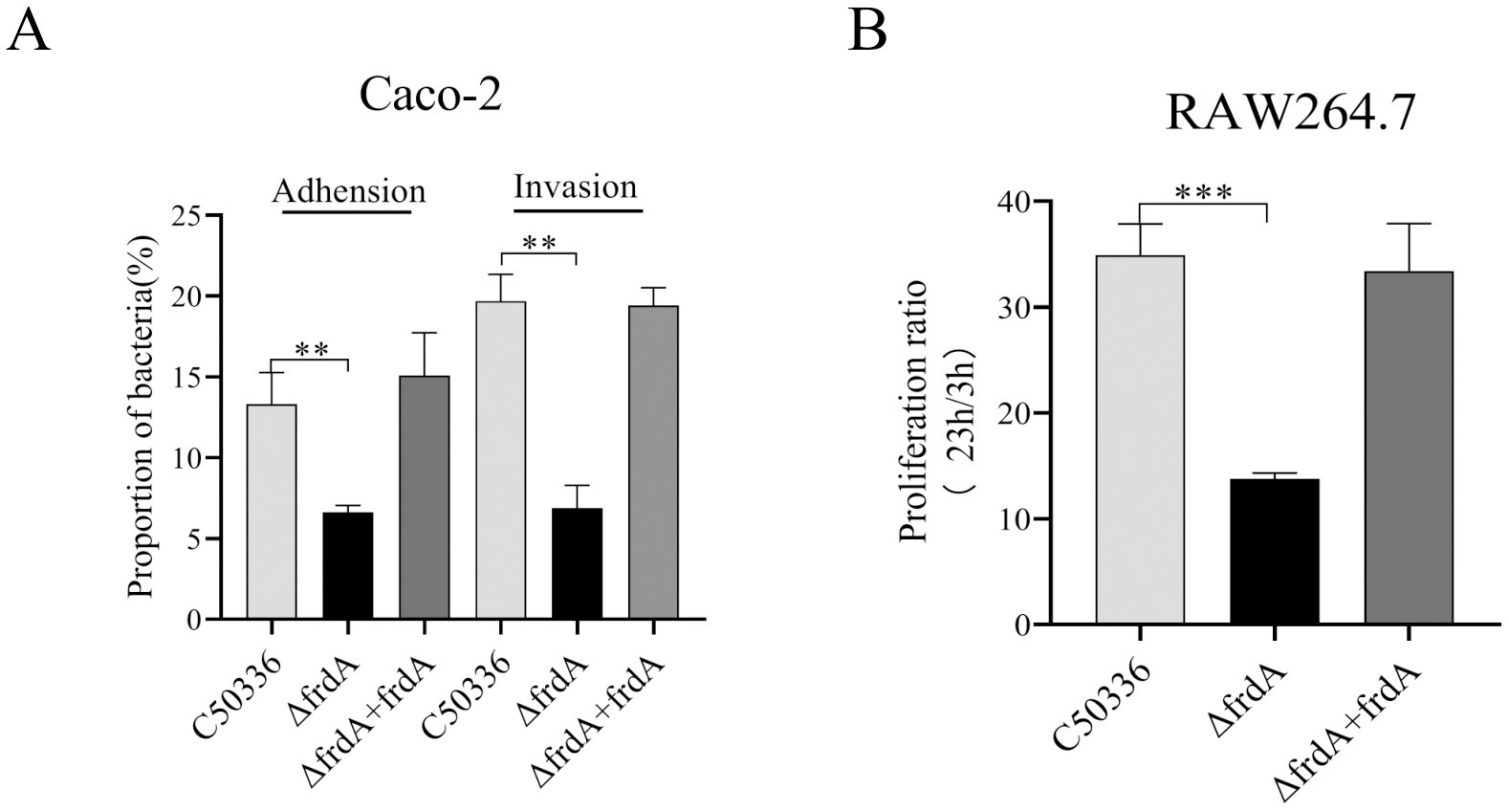
**(A)** Adhesion and invasion of bacteria in Caco-2 cells. **(B)** Intracellular survival in RAW264.7 cells. The data represents the average of 3 replicates (**p < 0.01, and ***p < 0.001).

The data showed that the Δ*frdA* displayed decreased adhesion and invasion to Caco-2 cells (Figure 5A) and impaired survival in macrophage RAW264.7 cells (Figure 5B). This indicates that the *frdA* gene is able to affect the adhesion, invasion, and intracellular survival ability of *S. enteritidis*.

### 3.6 The *frdA* gene knockout leads to attenuated virulence of *S. enteritidis*

The virulence of C50336 and Δ*frdA* was assessed by the mouse model via intraperitoneal injection. The mice that received injections of both strains began to show symptoms such as trembling, arched backs, crusted eyes, disheveled fur, and even death. The mice of the control group showed no abnormality. The deaths of mice were recorded for LD_50_ calculation. The LD_50_ of Δ*frdA* and C50336 were 7.94 × 10^7^ CFU and 1.26 × 10^6^CFU, respectively, with a 64-fold difference (Table 3).

**Table 3:** LD_50_ of C50336 and Δ*frdA* in KM mice.

**Strain**	**Inoculation dose (CFU/mouse)**	**No. of deaths/total no. of mice**	**LD50 (CFU)**
C50336	2 × 10^9^	10/10	
	2 × 10^8^	10/10	
	2 × 10^7^	10/10	1.26 × 10^6^
	2 × 10^6^	8/10	
	2 × 10^5^	0/10	
*ΔfrdA*	1 × 10^9^	10/10	
	1 × 10^8^	6/10	
	1 × 10^7^	0/10	7.94 × 10^7^
	1 × 10^6^	0/10	
	1 × 10^5^	0/10	

### 3.7 The *frdA* gene knockout leads to altered expression of numerous genes in *S. enteritidis*

**Figure 6:**
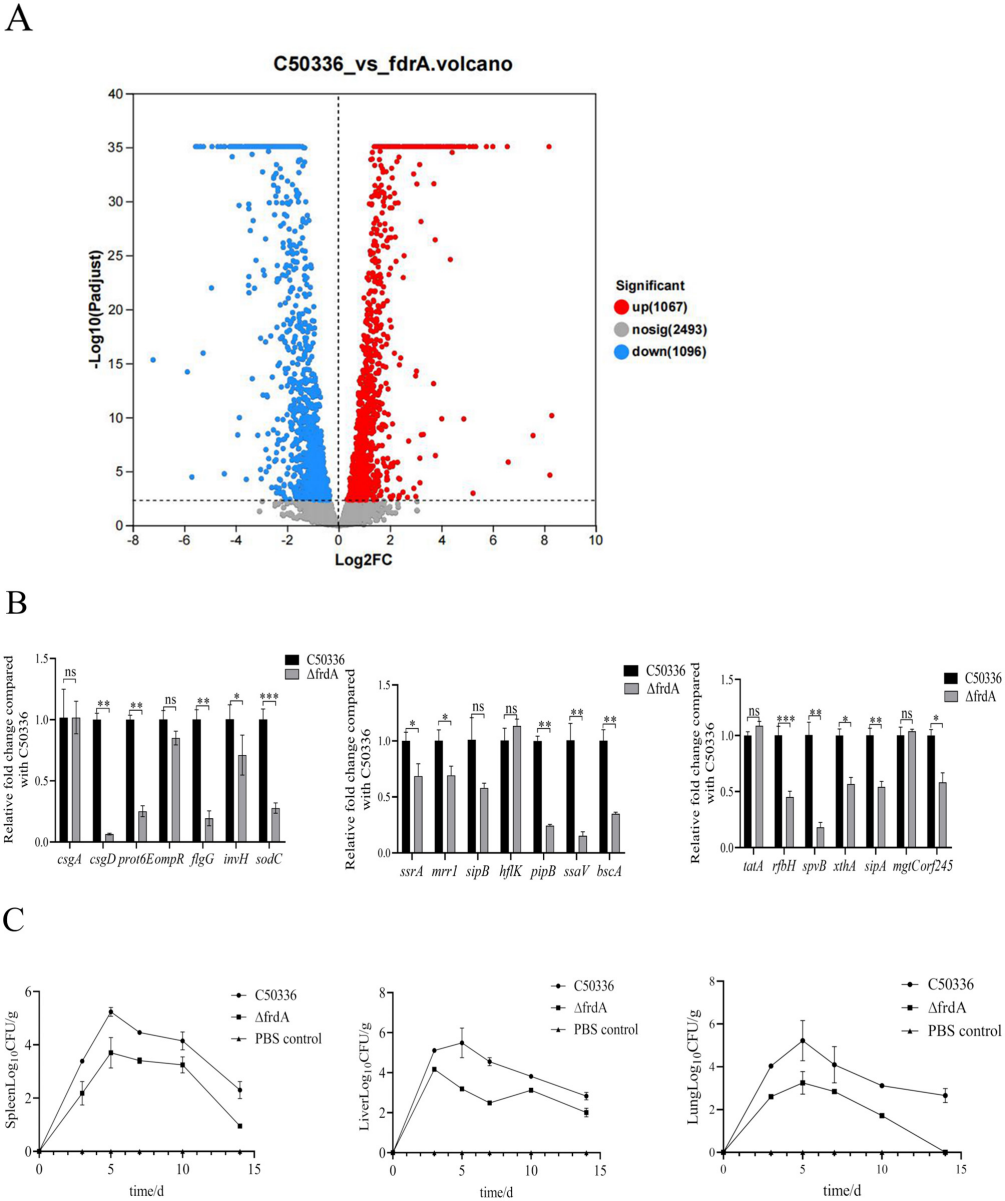
**(A)** Scatter plot of differentially expressed genes. **(B)** The expression levels of virulence genes in C50336 and Δ*frdA* were detected by using qPCR, with 16 S rRNA as the housekeeping gene. The data represents the average of 3 replicates (*p < 0.05, **p < 0.01, ***p < 0.001, ns means not significant). **(C)** Bacterial loads were determined in the spleen, liver and lungs of mice infected with C50336 and Δ*frdA*.

To further reveal the mechanism underlying FrdA's effect on *S. enteritidis*, a transcriptomic approach was employed to screen and analyze the differentially expressed genes after *frdA* deletion. A total of 2,163 DEGs were determined, with 1,067 up-regulated genes and 1,096 down-regualted genes (Figure 6A).

Among these DEGs, we focused on the those associated with bacterial virulence, and confirmed the expression of these genes using qPCR method. The expression of *csgD, prot6E, flgG, invH, sodC, ssrA, mrrl, pipB, ssaV, bscA, rfbH, spvB, sipA, xthA, or, f245* was significantly reduced (Figure 6B). This datas suggest that the *frdA* gene may regulate the virulence of *S. enteritidis* by modulating expression of multiple genes.

The bacterial burden of tissues and organs from C50336 and Δ*frdA* infected mice was assayed here. Although mice were inoculated with equal doses of C50336 and *frdA*, the bacterial loads of Δ*frdA* were significantly lower in the liver, spleen, and lungs than those of the wild-type strain at different time points (Figure 6C). To sum up above, all these results demonstrated that *frdA* deletion would lead to attenuation of *S. enteritidis*.

### 3.8 The Δ*frdA* gene provides a promising protection against *S. enteritidis*

**Figure 7:**
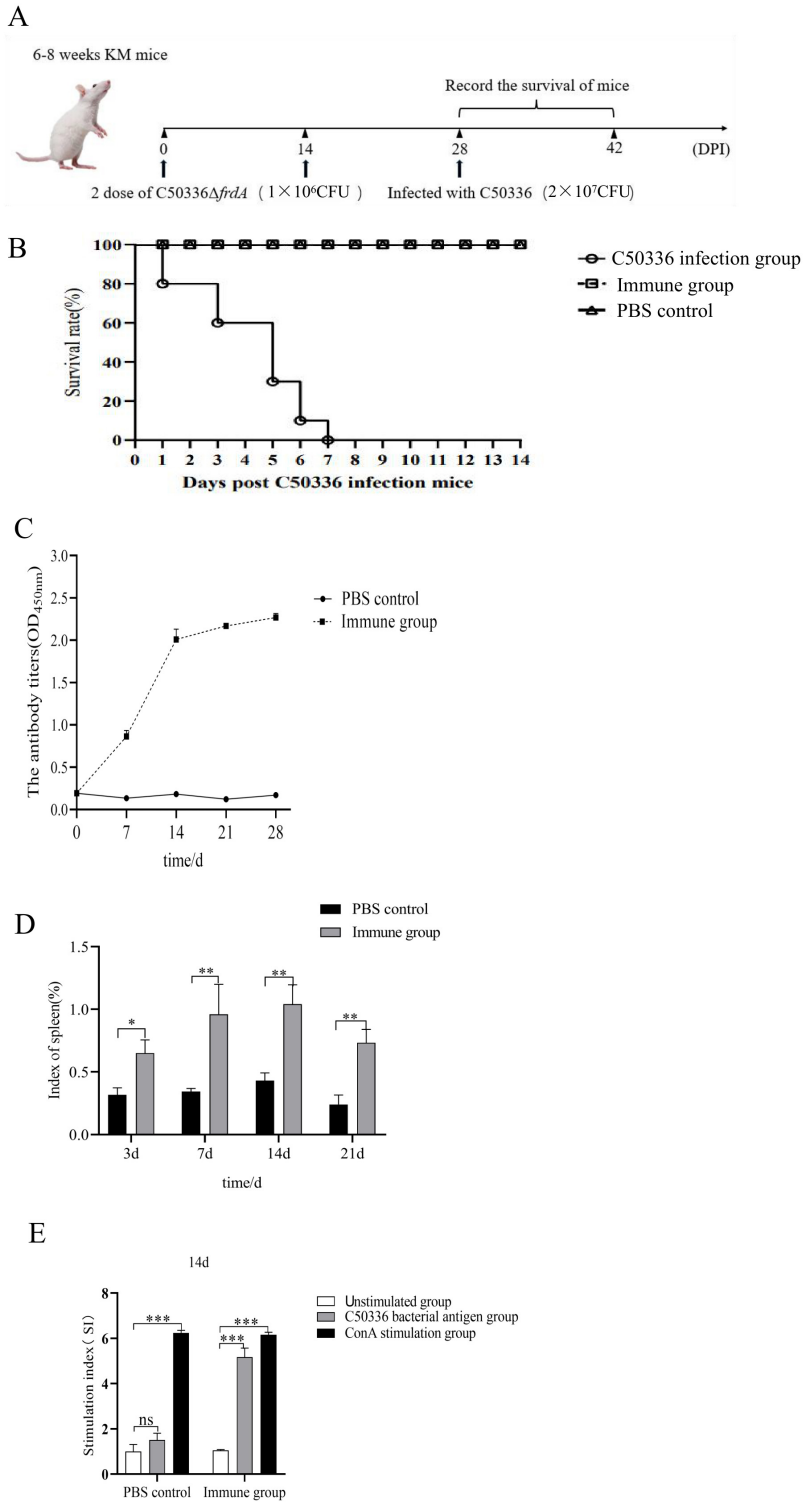
**(A)** Female KM mice (*n* = 10 per group) aged 6–8 weeks were orally immunized with the Δ*frdA* and orally immunized with a lethal dose of C50336 (5 × 10^6^ CFU/mouse) at 28 dpi. The survival rate of mice was monitored daily. **(B)** Survival curve. **(C)** Detection of antibody levels in mouse serum. **(D)** Spleen index of mice immunized from 7 days to 21 days. **(E)** Value-added capacity of mouse splenic lymphocytes after 14 dpi of immunization. (*p < 0.05, **p < 0.01, ***p < 0.001, and ns means not significant).

To determine the immunoprotective potential of Δ*frdA*, mice were challenged with a lethal dose of C50336 at 28 days post immunization with Δ*frdA* or PBS as control. The results showed (Figures 7A, B) that the mice that received Δ*frdA* immunization displayed a survival rate of 100% with mild and transient depression, while the mice from the unimmunized group developed typical symptoms of *S. enteritidis* infection and final death with a 100% mortality.

Mice serum was collected at different days post immunization, and the results of antibody detection showed the Δ*frdA* elicted IgG production at 7 days after immunization, and antibody levels were maintained at a high level after day 14 (Figure 7C), suggesting Δ*frdA* could induce effective humoral immunity.

The spleen indices of the mice were measured at different time post immunization, and the results showed that the spleen indices of the immunized group of mice were much higher than those of the control group (Figure 7D). For cellular immunity, mice splenocytes were harvested for lymphocyte proliferation assays 14 days post immunization.The SI index of the immunized group of mice was much higher than that of the unimmunized group (Figure 7E).

Taken together, the Δ*frdA* could elicit an effective immune response and provide promising protection against *S. enteritidis*.

## 4 Discussion

*S. enteritidis* is an important zoonotic pathogen with the ability to infect a wide range of animals, posing a significant threat (Wang et al., 2004). The bacterium develops the ability to modulate metabolism to adapt to the extreme environments to survive and cause infections. Although the association of TCA and bacterial virulence has been well-documented, as a complex system, the mechanism by which it affects *Salmonella* infections is not clear. Here we focus on FrdA, fumarate reductase of the TCA cycle. We found deletion of the *frdA* gene led to changes in multiple biological phenotypes of *S. enteritidis*, such as impaired tolerance to environmental stress, reduced motility, and decreased biofilm formation. Furthermore, knockout of *frdA* resulted in attenuated virulence in mice, with a 60 times increased LD_50_, accompanied by greatly decreased bacterial load in organs and down-regulated expression of multiple virulence genes. Moreover, we found that the *frdA* mutant, when taken as an immunogen, would induce an effective immune response and provide promising protection against *S. enteritidis* infection.

*S. enteritidis* is primarily transmitted through the digestive tract and ultimately invades a variety of cells, and it must overcome harsh environments (Li et al., 2023), such as extreme pH in the gastrointestinal tract. Within cells, oxidative stress, acidic stress, and alkaline stress are also essential limitations to the long-term survival of *Salmonella*. Many virulence-related genes have been reported to be involved in stress tolerance in *Salmonella* spp. (Sebkova et al., 2008; Das et al., 2020). In this study, we found that the *frdA* mutant displayed a weakened tolerance to environmental stress and may affect the infection progress.

The biofilm formation is another adaptive mechanism for *Salmonella* to cope with a stressful environment (Høiby et al., 2015). By wrapping the bacterium in a structure composed of a polysaccharide matrix, fibronectin, and lipoproteins, the biofilm can effectively protect the bacterium from the killing of antimicrobial peptides and antibiotics and promote bacterial adhesion and infection establishment (Ortega-Pena et al., 2020). The relationship between biofilm formation and bacterial pathogenicity has also been revealed in some studies about virulence genes or live vaccines. For example, *aroA* and *aroD* have been reported to affect biofilm formation in *S. enteritidis* (Malcova et al., 2009; Hewawaduge et al., 2023). In this study we got similar results that deletion of *frdA* decreased biofilm formation of *S. enteritidis* by affecting pruduction of curli and cellulose, the main components of biofilm.

The flagellum functions in bacterial motility and signaling, and plays pivotal roles in bacterial pathogenesis. A previous study reported that *frdA* can bind to FliG proteins to affect flagellar rotation (Koganitsky et al., 2019). This finding provides an explanation for our results, that the bacterial motility was significantly reduced after deletion of *frdA* from the *S. enteritidis*.

Adhesion and invasion to intestinal epithelial cells is the first step of infection by *S. enteritidis*. After crossing the intestinal barrier, *S. enteritidis* is engulfed by phagocytes and disseminates by following the phagocyte's migration (Dai et al., 2024). In this study, we also found decreased adhesion and invasion to Caco-2 cells and the delayed survival of *S. enteritidis* in macrophages RAW264.7 after the deletion of *frdA*.

Based on the effects of FrdA on multiple bacterial phenotypes, we hypothesized that it is closely involved in the virulence of *S. enteritidis*. As expected, we found that FrdA deficiency caused a significant decrease in *S. enteritidis* virulence, accompanied by an apparently decreased bacterial load in the major target organs, liver and spleen. Significantly decreased expression of virulence genes in *frdA*-deficient strains, such as *flgG* and *invH*, also supported the close relationship between FrdA and virulence of *S. enteritidis*.

Although the correlation between the TCA cycle and *Salmonella* virulence has been reported, it is limited to simply explain FrdA mediating *Salmonella* infection from this perspective. *Salmonella* undergoes a switch from aerobic to anaerobic metabolism during infection, whereas anaerobic metabolism dominates during *in vivo* infection, especially intracellular infection, without the involvement of the oxidative TCA cycle (Himpsl et al., 2020). Fumarate reductase is strongly induced in anaerobic metabolism and mediates the energy supply of bacteria under low-oxygen conditions by providing an alternative electron acceptor for the respiratory chain (Encheva et al., 2009). This may be a significant contributor to the reduced virulence of *Salmonella* from FrdA deletion.

Due to the intracellular parasitism, inactivated vaccines against *Salmonella* are not satisfactory, and many deletion strains based on virulence and metabolism-related genes have displayed better performance. In this study, Δ*frdA*, when used as an immunogen, was able to stimulate strong immune response in mice and displayed reliable protection against *S. enteritidis* infection, suggesting that Δ*frdA* is a good vaccine candicate. The limitation of this study lies in the use of intraperitoneal injection to assess the immunoprotective properties of FrdA-deficient strain, which differs from the natural route of infection of *S. enteritidis*, and needs confirmation by further oral immunization.

In summary, we demonstrated that *frdA* is involved in *Salmonella* pathogenicity and is an excellent vaccine target.

## Data Availability

The data that support the findings of this study are openly available in Science Data Bank. The DOI is 10.57760/sciencedb.22971.

## References

[B1] Altinok I. Capkin E. Karsi A. (2015). Succinate dehydrogenase mutant of *Listonella anguillarum* protects rainbow trout against vibriosis. Vaccine 33, 5572–5577. 10.1016/j.vaccine.2015.09.003 26382599

[B2] Arora G. Chaudhary D. Kidwai S. Sharma D. Singh R. (2018). CitE enzymes are essential for *Mycobacterium tuberculosis* to establish infection in macrophages and guinea pigs. Front. Cell. Infect. Microbiol. 8, 385. 10.3389/fcimb.2018.00385 30460206 PMC6232273

[B3] Arunima A. Swain S. K. Ray S. Prusty B. K. Suar M. (2020). RpoS-regulated SEN1538 gene promotes resistance to stress and influences *Salmonella enterica* serovar *enteritidis* virulence. Virulence 11, 295–314. 10.1080/21505594.2020.1743540 32193977 PMC7161692

[B4] Chen Q. Yu Y. Xu Y. Quan H. Liu D. Li C.. (2024). Salmonella typhimurium alters galactitol metabolism under ciprofloxacin treatment to balance resistance and virulence. *J. Bacteriol*. 206;e0017824. 10.1128/jb.00178-24 39082861 PMC11340313

[B5] Dai Y. Zhang M. Liu X. Sun T. Qi W. Ding W.. (2024). Salmonella manipulates macrophage migration via SteC-mediated myosin light chain activation to penetrate the gut-vascular barrier. EMBO J. 43, 1499–1518. 10.1038/s44318-024-00076-7 38528181 PMC11021425

[B6] Das S. Ray S. Arunima A. Sahu B. Suar M. A. (2020). ROD9 island encoded gene in *Salmonella enteritidis* plays an important role in acid tolerance response and helps in systemic infection in mice. Virulence 11, 247–259. 10.1080/21505594.2020.1733203 32116124 PMC7051147

[B7] Ding Y. Liu X. Chen F. Di H. Xu B. Zhou L.. (2014). Metabolic sensor governing bacterial virulence in Staphylococcus aureus. Proc. Natl. Acad. Sci. USA. 111, E4981–E4990. 10.1073/pnas.1411077111 25368190 PMC4246259

[B8] Dong H. Peng D. Jiao X. Zhang X. Geng S. Liu X.. (2011). Roles of the spiA gene from *Salmonella enteritidis* in biofilm formation and virulence. Microbiology. 157, 1798–1805. 10.1099/mic.0.046185-0 21415117 PMC3167914

[B9] Encheva V. Shah H. N. Gharbia S. E. (2009). Proteomic analysis of the adaptive response of *Salmonella enterica* serovar *typhimurium* to growth under anaerobic conditions. Microbiology 155(Pt 7), 2429–2441. 10.1099/mic.0.026138-0 19389776

[B10] Hewawaduge C. Senevirathne A. Sivasankar C. Lee J. H. (2023). The impact of lipid A modification on biofilm and related pathophysiological phenotypes, endotoxicity, immunogenicity, and protection of *Salmonella typhimurium*. Vet. Microbiol. 282:109759. 10.1016/j.vetmic.2023.109759 37104940

[B11] Himpsl S. D. Shea A. E. Zora J. Stocki J. A. Foreman D. Alteri C. J.. (2020). The oxidative fumarase FumC is a key contributor for *E. coli* fitness under iron-limitation and during UTI. PLoS Pathog. 16:e1008382. 10.1371/journal.ppat.1008382 32106241 PMC7064253

[B12] Høiby N. Bjarnsholt T. Moser C. Bassi G. L. Coenye T. Donelli G.. (2015). ESCMID guideline for the diagnosis and treatment of biofilm infections 2014. Clin. Microbiol. Infect. 21, S1–S25. 10.1016/j.cmi.2014.10.024 25596784

[B13] Kim H. Y. Lee G. Y. Thompson A. J. McBride R. Fu D. J. Paulson J. C.. (2025). Molecular basis of the hepatobiliary tropism of typhoid toxin promoting *Salmonella* pathogenicity. Sci Adv. 11:eadt2040. 10.1126/sciadv.adt2040 40479051 PMC12143353

[B14] Koganitsky A. Tworowski D. Dadosh T. Cecchini G. Eisenbach M. A. (2019). Mechanism of modulating the direction of flagellar rotation in bacteria by fumarate and fumarate reductase. J. Mol. Biol. 431, 3662–3676. 10.1016/j.jmb.2019.08.001 31412261 PMC6733631

[B15] Li W. Ren Q. Ni T. Zhao Y. Sang Z. Luo R.. (2023). Strategies adopted by Salmonella to survive in host: a review. Arch Microbiol. 205:362. 10.1007/s00203-023-03702-w 37904066

[B16] Malcova M. Karasova D. Rychlik I. (2009). aroA and aroD mutations influence biofilm formation in *Salmonella enteritidis*. FEMS Microbiol. Lett. 291, 44–49. 10.1111/j.1574-6968.2008.01433.x 19054077

[B17] Mitosch K. Beyss M. Phapale P. Drotleff B. Nöh K. Alexandrov T.. (2023). A pathogen-specific isotope tracing approach reveals metabolic activities and fluxes of intracellular *Salmonella*. PLoS Biol. 21:e3002198. 10.1371/journal.pbio.3002198 37594988 PMC10468081

[B18] Nguyen T. K. Bui H. T. Truong T. A. Lam D. N. Ikeuchi S. Ly L. K. T.. (2021). Retail fresh vegetables as a potential source of *Salmonella* infection in the Mekong Delta, Vietnam. Int. J. Food Microbiol. 2021:341. 10.1016/j.ijfoodmicro.2021.109049 33493824

[B19] Noster J. Hansmeier N. Persicke M. Chao T.-C. Kurre R. Popp J.. (2019a). Blocks in tricarboxylic acid cycle of *Salmonella enterica* cause global perturbation of carbon storage, motility, and host-pathogen interaction. mSphere 4:e00796–e00719. 10.1128/mSphere.00796-19 31826974 PMC6908425

[B20] Noster J. Persicke M. Chao T.-C. Krone L. Heppner B. Hensel M.. (2019b). Impact of ros-induced damage of tca cycle enzymes on metabolism and virulence of *Salmonella enterica* serovar *typhimurium*. Front. Microbiol. 10:762. 10.3389/fmicb.2019.00762 31105651 PMC6491894

[B21] Ortega-Pena S. Martínez-Garca S. Rod-Riguez-Martinez S. Cancino-Diaz M. E. Cancino-Diaz J. C. (2020). Overview of *Staphylococcus epidermidis* cell wall-anchored proteins: potential targets to inhibit biofilm formation. Mol Bio Rep. 47, 771–784. 10.1007/s11033-019-05139-1 31642039

[B22] Park S. Jung B. Kim E. Hong S.-T. Yoon H. Hahn T.-W. (2020). *Salmonella typhimurium* lacking YjeK as a candidate live attenuated vaccine against invasive *Salmonella* infection. Front. Immunol. 11:1277. 10.3389/fimmu.2020.01277 32655567 PMC7324483

[B23] Sebkova A. Karasova D. Crhanova M. Budinska E. Rychlik I. (2008). Aro mutations in *Salmonella enterica* cause defects in cell wall and outer membrane integrity. J. Bacteriol. 190, 3155–3160. 10.1128/JB.00053-08 18310348 PMC2347392

[B24] Simm R. Ahmad I. Rhen M. Le Guyon S. Römling U. (2014). Regulation of biofilm formation in *Salmonella enterica* serovar *typhimurium*. Fut. Microbiol. 9, 1261–1282. 10.2217/fmb.14.88 25437188

[B25] Steinsiek S. Frixel S. Stagge S. Sumo Bettenbrock K. (2011). Characterization of *E. coli* MG1655 and *frdA* and sdhC mutants at various aerobiosis levels. J Biotechnol. 154, 35–45. 10.1016/j.jbiotec.2011.03.015 21458504

[B26] Sun Y. Zhang Y. Zhao T. Luan Y. Wang Y. Yang C.. (2023). Acetylation coordinates the crosstalk between carbon metabolism and ammonium assimilation in *Salmonella enterica*. EMBO J. 42:e112333. 10.15252/embj.2022112333 37183585 PMC10308350

[B27] Tchawa Yimga M. Leatham M. P. Allen J. H. Laux D. C. Conway T. Cohen P. S.. (2006). Role of gluconeogenesis and the tricarboxylic acid cycle in the virulence of *Salmonella enterica* serovar *typhimurium* in BALB/c mice. Infect. Immun. 74, 1130–1140. 10.1128/IAI.74.2.1130-1140.2006 16428761 PMC1360343

[B28] Wang M. Q. Ran L. Wang Z. T. Li Z. (2004). Active surveillance of food-borne pathogens and their drug resistance in China in 2001. Health Res. 33, 49–54. 10.1186/s12889-025-23439-z 15098478

[B29] Westerman T. L. McClelland M. Elfenbein J. R. (2021). YeiE Regulates motility and gut colonization in *Salmonella enterica* serotype *typhimurium*. MBio 12, e03680–20. 10.1128/mBio.03680-20 34098734 PMC8262846

[B30] Yao X. Gao J. Wang L. Hou X. Ge L. Qin X.. (2024). Cananga oil inhibits *Salmonella* infection by mediating the homeostasis of purine metabolism and the TCA cycle. J Ethnopharmacol. 325:117864. 10.1016/j.jep.2024.117864 38325671

[B31] Zeng F. Pang H. Chen Y. Zheng H. Li W. Ramanathan S.. (2020). First succinylome profiling of vibrio alginolyticus reveals key role of lysine succinylation in cellular metabolism and virulence. Front. Cell. Infect. Microbiol. 10:626574. 10.3389/fcimb.2020.626574 33614530 PMC7892601

[B32] Zhang L. Wu T. Wang F. Liu W. Zhao G. Zhang Y.. (2024). CheV enhances the virulence of *Salmonella enteritidis*, and the Chev-deleted *Salmonella* vaccine provides immunity in mice. BMC Vet. Res. 20:100. 10.1186/s12917-024-03951-x 38468314 PMC10926574

[B33] Zhang Z. Du W. Wang M. Li Y. Su S. Wu T.. (2020). Contribution of the colicin receptor CirA to biofilm formation, antibotic resistance, and pathogenicity of *Salmonella enteritidis*. J. Basic Microbiol. 60, 72–81. 10.1002/jobm.201900418 31737922

[B34] Zhao G. Duan W. Zhang L. Sun W. Liu W. Zhang X.. (2024). The peptidoglycan-associated lipoprotein gene mutant elicits robust immunological defense in mice against *Salmonella enteritidis*. Front. Microbiol. 15:1422202. 10.3389/fmicb.2024.1422202 38903796 PMC11188350

[B35] Zhao P. Xiao C. Xuan M. Yan S. Yu X. Li W.. (2025). Exploring the potential of coix seeds to mitigate high humidity-induced gut inflammation via microbiota and metabolite modulation. J. Inflamm. Res. 18, 10193–10211. 10.2147/JIR.S524947 40761380 PMC12318861

[B36] Zhou N. Ding Y. He T. Sun Y. Chen H. Huang M.. (2025). Characterization and protective efficacy of a *Salmonella typhimurium* ATCC 14028 sptP mutant as a live attenuated vaccine candidate. Vaccines 13:150. 10.3390/vaccines13020150 40006697 PMC11860608

